# Modeling and Identification for Vector Propulsion of an Unmanned Surface Vehicle: Three Degrees of Freedom Model and Response Model

**DOI:** 10.3390/s18061889

**Published:** 2018-06-08

**Authors:** Dongdong Mu, Guofeng Wang, Yunsheng Fan, Xiaojie Sun, Bingbing Qiu

**Affiliations:** School of Marine Electrical Engineering, Dalian Maritime University, Dalian 116026, China; ddmu.phd@gmail.com (D.M.); yunsheng@dlmu.edu.cn (Y.F.); xjsun.phd@gmail.com (X.S.); bbqiu.dmu@gmail.com (B.Q.)

**Keywords:** modeling, identification, vector propulsion, unmanned surface vehicle, field experiment, sensors, course keeping

## Abstract

This paper presents a complete scheme for research on the three degrees of freedom model and response model of the vector propulsion of an unmanned surface vehicle. The object of this paper is “Lanxin”, an unmanned surface vehicle (7.02 m × 2.6 m), which is equipped with a single vector propulsion device. First, the “Lanxin” unmanned surface vehicle and the related field experiments (turning test and zig-zag test) are introduced and experimental data are collected through various sensors. Then, the thrust of the vector thruster is estimated by the empirical formula method. Third, using the hypothesis and simplification, the three degrees of freedom model and the response model of USV are deduced and established, respectively. Fourth, the parameters of the models (three degrees of freedom model, response model and thruster servo model) are obtained by system identification, and we compare the simulated turning test and zig-zag test with the actual data to verify the accuracy of the identification results. Finally, the biggest advantage of this paper is that it combines theory with practice. Based on identified response model, simulation and practical course keeping experiments are carried out to further verify feasibility and correctness of modeling and identification.

## 1. Introduction

Ship motion control is an important field that combines navigation science and technology. Its ultimate purpose is to improve the level of ship automation and intelligence, and to ensure the safety, economy and comfort of a ship’s navigation [1,2,3]. Meanwhile, with the continuous development of technology and the extension of the field of ship application, the model of a traditional ship is developing towards miniaturization, speediness and intellectualization, and a new surface carrier has been developed—Unmanned Surface Vehicle (USV) [4,5,6,7]. To allow better maneuverability, the propulsion system of a USV has higher requirements. The vector propulsion system (POD, waterjet and outboard servos, etc.) is a new propulsion device in the field of ship propulsion, which has proven to be more efficient than conventional propeller-rudders [8,9,10].

The mathematical model of a ship is the basis for realizing intelligent control and maneuverability prediction. The history of its development can be traced back to 1946. Davidson and Schiff applied the knowledge of rigid dynamics and fluid mechanics for the first time to propose a maneuvering motion model. After continuous exploration and development, two kinds of mathematical models of ship motion have resulted. One is the Abkowitz model, which is also called the global model. Its essence is to consider the hull, propeller and rudder as a whole and carry out Taylor series expansion at the equilibrium position of the fluid acting on the hull [11,12]; the other is a separate model proposed by a ship manoeuvring mathematical model group (MMG), also known as the MMG model [13,14]. Its essence is to decompose the hydrodynamic forces acting on the bare hull, the thrust of the propeller and the force of the rudder according to the physical meaning, and then to consider the interference between them. In general, the separation modeling theory is more convenient for theoretical analysis than the global modeling theory; therefore, the MMG model is employed to analyze the model structure of vector propulsion USV in this paper. Meanwhile, based on the actual situation, we designed the method for obtaining model parameters. The empirical formula method was employed to obtain the thruster (it is produced by the propeller) model. Based on field experimental data, the model parameters of three degrees of freedom (DOF) model, response model and thruster servo model were obtained by system identification [15,16,17].

In the past few decades, various results have been shown in literature [18,19,20,21]. Fossen et al. put forward a method of modeling and identification for a fully driven model supply ship (mass is 23.8 kg, length is 1.255 m, breadth is 0.29 m), the classic CyberShip II in the Marine Cybernetics Laboratory at Norwegian University of Science and Technology, which is widely used in the study of all kinds of ship motion control [22]. The system identification techniques used in [23] included the extended Kalman filter (EKF) and the constrained least-squares method. In [24], a novel identification scheme for non-linear manoeuvring models based on two steps was proposed. Sonnenburg et al. described planar motion modeling for an USV, including a comparative evaluation of several experimentally identified models over a wide range of speeds and planing conditions [25]. On the premise of obtaining full-scale trial data, a new transformed multi-innovation least squares algorithm was used to identify the model parameters of a four DOF model for a YUKUN ship [26]. In [27], a system-based method was hired to investigate a four DOF ship maneuvering motion in calm water for the ONR tumblehome model. However, most of the literature, including the above, have researched ordinary propeller-rudder propulsion ships. Motivated by the above-mentioned observations, the thruster model, three DOF model and response model, which are obtained using the empirical formula method and system identification method, were established for a vector propulsion USV. First, field experiment data were collected through the related sensors. Then, modeling, identification, and validation work were performed separately. Finally, numerical simulation and real ship experiments for the course keeping of USVs were performed. The main contributions of this paper can be summarized as follows:

(1) According to the force analysis and hypothesis, it is proved that the three degrees of freedom (DOF) model of vector propulsion of USVs is an underactuated system, and its response model still conforms to the classic Norrbin model structure. In addition, this conclusion can be generalized to general vector propulsion ships.

(2) The empirical formula method and system identification method are combined to get the parameters of the three DOF model and response model.

(3) The greatest advantage of this paper is the connection between theory and practice. The actual course keeping field experiment is carried out to further verify the correctness of the modeling and identification results.

The rest of this paper is organized as follows. In Section 2, the Lanxin USV and field experiments are introduced. Section 3 shows the models of thruster thrust and servo. The modeling process of the three DOF model and the response model is shown in Section 4. In Section 5, the identification and validation results are displayed. In Section 6, combining theory with practice, we carry out a real ship course keeping experiment. Section 7 contains the conclusions.

## 2. Field Experiment of Data Acquisition

In this section, Lanxin USV is first introduced, and then data is collected from field experiments by related sensors.

### 2.1. Lanxin USV

**Figure 1:**
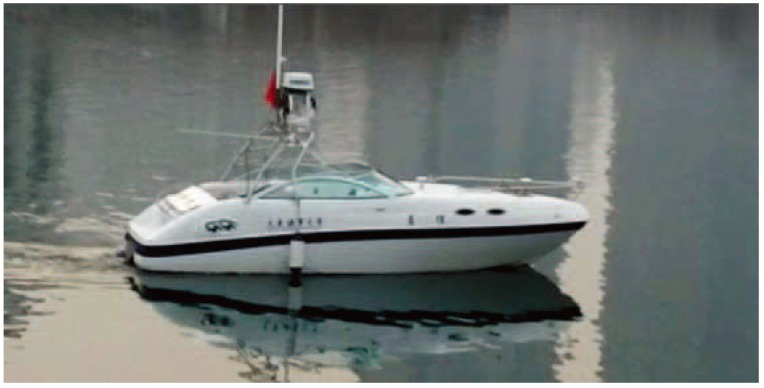
Lanxin USV.

Lanxin USV is the foundation of the field experiment, which is mainly composed of a power propulsion system, an automatic control system and an information collection system, a communication system, and an image acquisition system. The appearance of the USV is shown in Figure 1, and its specific parameters are displayed in Table 1.

### 2.2. Vector Propulsion System

**Figure 2:**
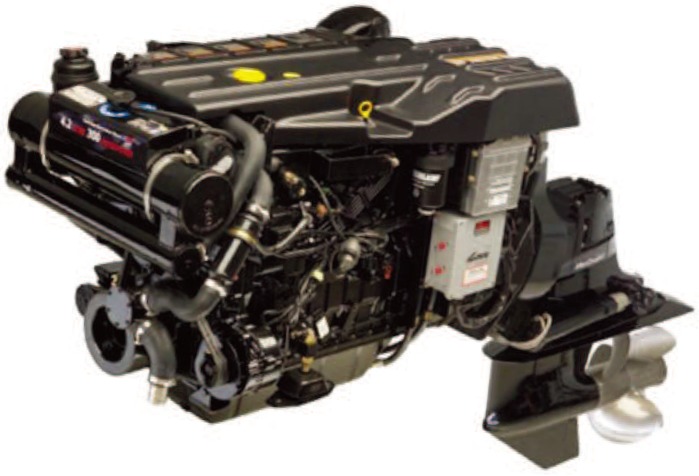
Vector propulsion system.

The dynamic propulsion system guarantees a continuous output of the USV power and is an important component to ensure the performance of a USV. The dynamic propulsion system is equipped with an electric displacement gasoline engine with a displacement of 5 liters, and its maximum output power can reach to 260 HP. The stern machine is equipped with an 0.46 meter diameter three leaf propeller (For the Lanxin USV, the propeller is its thruster), which can ensure a greater transmission ratio and low speed navigation stability. Vector propulsion system is shown in Figure 2.

Vector propulsion is one of the most promising new technologies in the field of ship propulsion, and has the characteristics of improving the efficiency and maneuverability of the ship. The vector thruster can rotate ±35° around the axis, and can achieve maximum thrust in any direction within the scope of +35° and −35°.

### 2.3. Platform of Sensor Network

**Figure 3:**
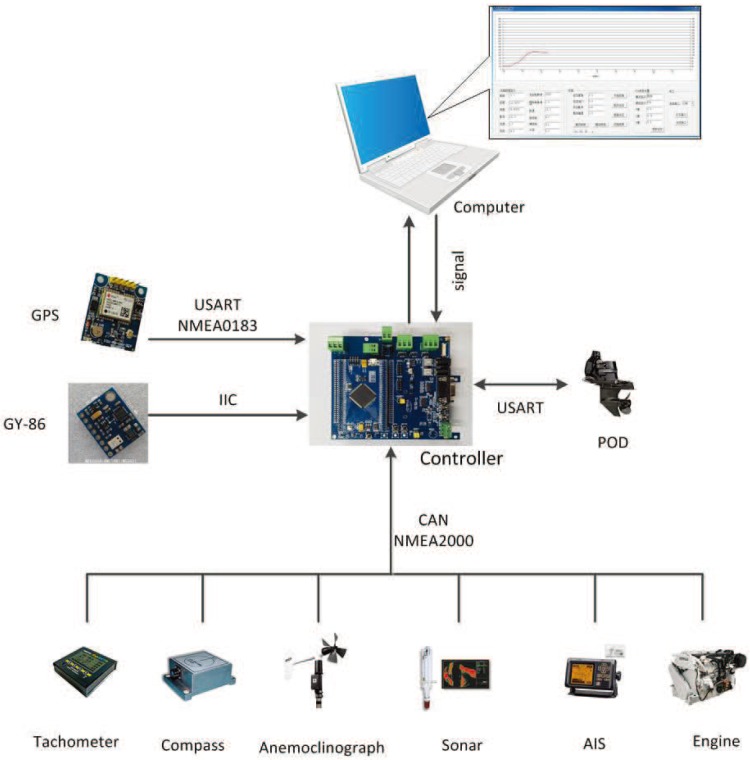
Multi-sensor structure.

To achieve precise control, the controller first needs to obtain the motion state of the USV and the real-time information of the surrounding environment through a variety of detection sensors. The multi-sensor system of the Lanxin USV includes the following three parts: a GY-86 attitude detection sensor (Simrad, Egersund, Norway), a global position system (GPS) navigation sensor (Simrad, Egersund, Norway) and a yacht equipment network. The attitude detection sensor is communicated with the microcontroller through the inter integrated circuit (IIC) protocol. It mainly provides attitude information about the USV, such as the rolling rate, pitching rate and course angle. The GPS navigation sensor is connected with a microcontroller through a serial port. The communication protocol adopted is the national marine electronics association (NMEA)0183 protocol, which mainly provides latitude and longitude information. The network system of yacht equipment is communicated with the microcontroller through the controller area network (CAN) bus. The communication protocol adopts the NMEA2000 protocol, which mainly provides the state information and information about the surrounding environment of the USV, such as the driving speed, the rotating speed of propeller, the water depth, the power supply voltage and so on. The multi-sensor structure of a USV is shown in Figure 3.

(1) GY-86 Attitude Detection Sensor

The attitude sensor module uses three chips, MPU6050, HMC5883L and MS5611, and four sensors are mounted on board. MPU6050 integrates a microelectro mechanical systems (MEMS) gyroscope and a MEMS accelerometer. The magnetic field intensity sensor used is HMC5883L. The air pressure sensor used is MS5611. They are connected in series and are able to compensate for each other’s defects by using an algorithm.

(2) GPS Navigation Sensor

The GPS navigation sensor adopts the NEO-5Q main chip (U-blox, Zurich, Switzerland). The chip is a multi-functional and independent GPS module, which has the advantages of low cost and small volume. The communication between the GPS navigation module and the motion controller is the NMEA0183 protocol, and the NMEA0183 is now the standard protocol for unified GPS navigation equipment. It is the standard format made by the National Oceanic Association of the United States for marine electronic equipment.

(3) Yacht Equipment Network System

The network system is an important part of the state information collection of unmanned vehicles. The system interconnects the engine, sonar, compass and other craft equipment through the NMEA2000 protocol, and forms an efficient information sharing network. The computer terminal adopts microsoft foundation classes (MFC) visual programming, which can monitor and display various information about the USV in real-time and stores the collected data in the database. The controller adopts the digital modular design based on STM32 as the microcontroller. It mainly completes functions such as data acquisition, information release, and steering control. The controller has a variety of sensor interfaces and integrates the required communication protocols to obtain real-time data from the boat-borne sensor network.

### 2.4. Field Experiment

The specific contents of the field experiment included the turning test and the zig-zag test. Using a 10° turning test and 10°/10° zig-zag test as examples, their specific processes are as follows:

(1) Turning test: keeping the speed unchanged, rotate the propulsion angle to 10° and wait for the USV to rotate steadily.

(2) Zig-zag test: keeping the speed unchanged, rotate the propulsion angle to 10°, and when the course angle changes to 10°, push the propulsion angle to −10°. After that, until the course angle changes to −10°, rotate the propulsion angle to 10°. Repeat the above steps several times.

**Remark** **1.** *For a traditional propeller-rudder propulsion ship, the direction of navigation can be changed by changing its rudder angle. However, the vector propulsion USV has no rudder, so we call it a propulsion angle.*

**Remark** **2.** *The force produced by the propeller when it is rotating clockwise or counterclockwise has different effects on a USV, and the counterclockwise rotation of the propeller leads to reversing, which is not within the scope of this paper.*

**Figure 4:**
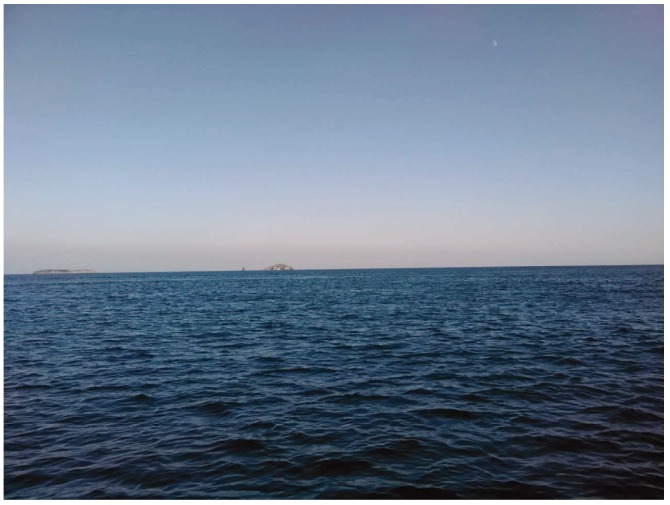
The sea state of the field experiment.

In this subsection, the contents of the field experiment are described in detail. In order to reduce the influence of external disturbance on the accuracy of the collected data, field experiments are conducted in a relatively calm sea state. The sea state is shown in Figure 4.

The environment and content of the field experiment:

(1) Experimental sea area: nearby waters (longitude: 121.5548, latitude: 38.8612).
(2) Sea state: one-level marine conditions. The sea surface was quite calm, and the waves were 0–0.1 m high.
(3) Weather: the weather was fine and the sea breeze was about a one-level northeasterly wind.
(4) The driving speed of USV: this was kept at around 10 knots (corresponding to this, the engine speed was about 2800/min).
(5) The contents of the record were the driving speed, *V*, course angle, ψ, rotating speed of the propeller, *n*, surge velocity, *u*, sway velocity, *v*, and yaw rate, *r*.
(6) Sampling frequency: 0.02 s.
(7) The specific contents of the field experiment were the turning test (10°, 15°, 25°, 35°) and the zig-zag test (10°/10°).

**Remark** **3.** *The maximum speed of the Lanxin USV is 30 knots. If the driving speed is too fast, the driver’s safety cannot be guaranteed, but slow speeds cannot fully explore the manipulation characteristics of the USV. So, in general, the driving speed of a USV is chosen to be kept around 10 knots.*

## 3. Thruster Thrust Model and Servo Model

### 3.1. Thruster Thrust Model

The vectorial thrust generated by the thruster (propeller) can be decomposed into two forces: the longitudinal force to keep the USV moving forward and the lateral force moment to change the direction of the USV. In essence, the latter is actually the effect of the rudder. To some extent, this vector propulsion device simplifies the traditional propeller rudder, especially for small ships represented by USVs, which greatly improves the propulsion efficiency. Meanwhile, the vectorial propulsion system has a more concise mechanical structure, which is more suitable for smaller USVs.

According to [28], the thrust mathematical model of the vector thruster can be expressed as

(1) $$\begin{array}{c} T=\left(1-t_{P}\right)\rho n^{2}{D_{P}}^{4}K_{T}\left(J_{p}\right), \end{array}$$

where $t_{P}$ represents the coefficient of the thrust reduction, ρ indicates the density of the sea water, *n* is the rotating speed of the propeller, $D_{p}$ is the diameter of the propeller, $K_{T}\left(J_{P}\right)$ is a propeller thrust coefficient, and $J_{P}$ is the advance coefficient.

#### 3.1.1. Thrust Reduction Factor ($t_{P}$)

The increase in hull resistance caused by propellers under working conditions is called an increase of resistance, which is represented by ΔR. If the propulsive force generated by the propeller under working conditions is *P*, part of the thrust will be used to overcome the resistance *R* of the forward hull, and another part will be used to overcome the resistance increase, ΔR. Thus, it can be seen that only part of the (P−ΔR) is used to push the USV forward, so it is called the effective thrust, $P_{e}$. Customarily, ΔR is called the thrust reduction, which is expressed as ΔP. In other words, $P_{e}=R$ and ΔP=ΔR. Then, the coefficient of thrust reduction, $t_{P}$, can be obtained by ΔP and *P*. That is

(2) $$\begin{array}{c} t_{P}=\frac{\Delta P}{P}=\frac{P-P_{e}}{P}=\frac{P-R}{P}. \end{array}$$

The value of $t_{P}$ is determined by a variety of factors, including the shape of the USV, the size of the propeller, the loading load and so on. In this paper, Hollo’s formula for a single boat is used to estimate the $t_{P}$:

(3) $$\begin{array}{c} t_{P}=0.001979\frac{L}{\left(B-BC_{p1}\right)}+1.0585C_{10}-0.000524-0.1418\frac{{D_{P}}^{2}}{Bd}+0.0015C_{stern}, \end{array}$$

where *B* indicates the maximum transverse width of USV hull, *L* is the maximum longitudinal length of the USV hull, *d* represents the average draught depth of the USV. $C_{p1}$ is defined as $C_{p1}=1.45C_{P}-0.315-0.225L_{cb}$, where $C_{P}$ is the prismatic coefficient and $L_{cb}\approx 0.5L$. $C_{10}$ is determined by the ratio of the longitudinal and transverse widths of the USV, and $C_{stern}$ is a coefficient associated with the shape of the tail of the USV. Their specific definitions are shown in (4) and (5).

(4) $$\begin{array}{c} \left\{\begin{array}{c} L/B>5.2\;C_{10}=B/L\\ L/B\leq 5.2\;C_{10}=0.25-\frac{0.003328402}{B/L-0.134615385} \end{array}\right. \end{array}$$

(5) $$\begin{array}{c} \left\{\begin{array}{cc} V-\mathrm{section} & C_{stern}=-10\\ \mathrm{Conventional} & C_{stern}=0\\ U-\mathrm{section} & C_{stern}=+10 \end{array}\right. \end{array}$$

According to the actual situation of the Lanxin USV and the above formula, its thrust reduction factor is $t_{P}\approx 0.05$.

#### 3.1.2. Thrust Coefficient ($K_{T}$)

The propeller of the USV is an ordinary three-bladed paddle, and according to [25], the thrust coefficient, $K_{T}$, of a three-bladed series paddle can be expressed as

(6) $$\begin{array}{c} K_{T}=\sum_{i=0}^{n_{1}}{\sum_{j=0}^{n_{2}}A_{ij}\left(\frac{P}{D}\right)}^{i}J_{P}^{j}, \end{array}$$

where the advance coefficient is $J_{P}=\frac{\left(1-w_{p}\right)u}{nD_{P}}$.

$w_{p}$ is the propeller flow coefficient. According to the Bamier formula, the expression for $w_{p}$ is

(7) $$\begin{array}{c} w_{p}=0.165C_{b}^{x}\sqrt{\frac{\sqrt[{3}]{\nabla }}{D_{P}}}-\Delta w, \end{array}$$

where ∇ represents the drainage volume of the USV, and *x* is an exponent. When x=1, it is applicable to the middle line propeller; when x=2, it is applicable to the side propeller. Δw represents the modified value of the flow coefficient, which can be expressed as

(8) $$\begin{array}{c} \Delta w=\left\{\begin{array}{cc} 0.1\left(F_{n}-0.2\right), & F_{n}>0.2\\ 0, & F_{n}\leq 0.2, \end{array}\right. \end{array}$$

where $F_{n}$ is the Furude coefficient. $F_{n}=V/\sqrt{gL}$, and *g* is the acceleration of gravity. Based on the conditions of the field experiment in Section 2.2 and the above formula, one can deduce that $w_{p}\approx 0.04$.

Then, using Lagrange interpolation, the disk surface ratio is also taken into account as an influencing variable. Based on this, (6) can be rewritten as

(9) $$\begin{array}{c} K_{T}=\frac{K_{T0.5}\left(\theta -0.8\right)\left(\theta -1.1\right)}{0.18}+\frac{K_{T0.8}\left(\theta -0.5\right)\left(\theta -1.1\right)}{-0.09}+\frac{K_{T1.1}\left(\theta -0.5\right)\left(\theta -0.8\right)}{0.18}, \end{array}$$

where $K_{T0.5}$, $K_{T0.8}$, and $K_{T1.1}$ are thrust factors with disk surface ratios of 0.5, 0.8, and 1.1, respectively.

In the first quadrant, the propeller’s regression formula is expressed as

(10) $$\begin{array}{cc} K_{T} & =A_{00}+A_{01}J_{P}+A_{02}J_{P}^{2}+A_{10}\left(\frac{P}{D}\right)+A_{20}{\left(\frac{P}{D}\right)}^{2}+A_{30}{\left(\frac{P}{D}\right)}^{3}\\ & +A_{11}J_{P}\left(\frac{P}{D}\right)+A_{12}J_{P}^{2}\left(\frac{P}{D}\right)+A_{21}J_{P}{\left(\frac{P}{D}\right)}^{2}+A_{22}J_{P}^{2}{\left(\frac{P}{D}\right)}^{2}. \end{array}$$

Meanwhile, the coefficients of regression formulas for $K_{T0.5}$, $K_{T0.8}$ and $K_{T1.1}$ are shown in Table 2.

### 3.2. Servo Model

The traditional rudder servo model is considered to be a first order inertial link [28,29,30]. That is,

(11) $$\begin{array}{c} \dot{\delta }=-\frac{1}{T_{d}}\delta +\frac{1}{T_{d}}\delta_{d}, \end{array}$$

where $T_{d}$ is a time constant, $\delta_{d}$ is the target propulsion angle, δ is actual propulsion angle, and $\left|\delta \right|\leq 35{}^{\circ}$.

The servo system of the USV has a higher sensitivity and faster activity. After analyzing the real ship’s experimental data, we cannot think of the servo model as a first-order inertia link. It should be regarded as a two-order under-damped system.

(12) $$\begin{array}{c} \ddot{\delta }+2\zeta \omega_{n}\dot{\delta }+\omega_{n}^{2}\delta =K_{n}\omega_{n}^{2}\delta_{d} \end{array}$$

where $\omega_{n}$ is the natural frequency, ζ is the damping ratio, and $K_{n}$ is the magnification factor.

## 4. Modeling

### 4.1. Three DOF Model

**Figure 5:**
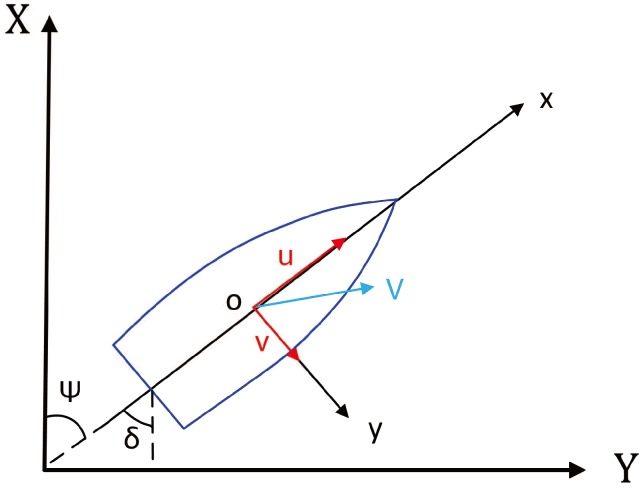
Schematic diagram of plane motion.

Control is the core of many problems, and the model is the basis of control. The design effect of the USV motion controller depends not only on the selected control algorithm, but also on the accuracy of the mathematical model. The complexity of the USV model is mainly manifested in the viscous hydrodynamic force of the hull, the control of the input force/torque, the high nonlinearity and coupling of the external disturbance. Therefore, the simplification of any structure in the model inevitably ignores some important characteristics of the system. The actual movement of the USV is very complex, with six DOF in general, including the surge velocity, sway velocity, heave velocity, yaw rate, rolling rate, and pitching rate. In previous research on the ship model, to simplify the research difficulty, the heave velocity, rolling rate and pitching rate have often been ignored. In other words, only consider the surge velocity *u*, the sway velocity *v* and the yaw rate *r* have been considered. The relationship between them is shown in Figure 5.

Before that, we first need to define some variables. $x_{g}$ is the distance from the center of the USV to the center of gravity, $X_{\dot{u}}$, $Y_{\dot{r}}$, $Y_{\dot{v}}$, $N_{\dot{r}}$, $I_{\dot{z}}$, $X_{u}$, $Y_{v}$, and $Y_{r}$, $N_{v}$, $N_{r}$ are the corresponding hydrodynamic coefficients, respectively.

The three degree of freedom dynamic equation established by the Lagrange’s mechanics theory can be expressed as

(13) $$\begin{array}{c} M\dot{\upsilon }+C\left(\upsilon \right)\upsilon +D\left(\upsilon \right)\upsilon =\tau , \end{array}$$

where $M=\left[\begin{array}{ccc} m-X_{\dot{u}} & 0 & 0\\ 0 & m-Y_{\dot{v}} & mx_{g}-Y_{\dot{r}}\\ 0 & mx_{g}-Y_{\dot{r}} & I_{z}-N_{\dot{r}} \end{array}\right]$, $\upsilon ={\left[u,v,r\right]}^{T}$, $C\left(\upsilon \right)=\left[\begin{array}{ccc} 0 & 0 & -\left(m-Y_{\dot{v}}\right)v-\left(mx_{g}-Y_{\dot{r}}\right)r\\ 0 & 0 & (m-X_{\dot{u}})u\\ \left(m-Y_{\dot{v}}\right)v+\left(mx_{g}-Y_{\dot{r}}\right)r & -(m-X_{\dot{u}})u & 0 \end{array}\right]$, $D\left(\upsilon \right)=\left[\begin{array}{ccc} -X_{u} & 0 & 0\\ 0 & -Y_{v} & -Y_{r}\\ 0 & -N_{v} & -N_{r} \end{array}\right]$ and $\tau ={\left[\tau_{u},\tau_{v},\tau_{r}\right]}^{T}$.

**Figure 6:**
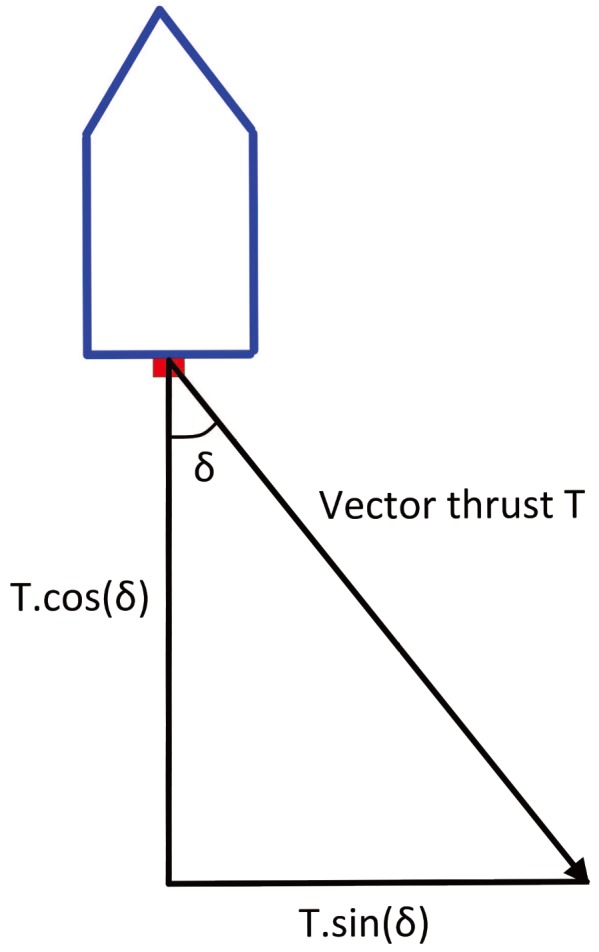
A schematic diagram of the vector thrust distribution.

*M* is called the inertia matrix, and C(υ) is the Coriolis/centripetal force matrix. D(υ) is the hydrodynamic damping matrix. $\tau_{u}$ is the longitudinal thrust, $\tau_{v}$ is the lateral thrust, and $\tau_{r}$ is the yaw moment. For the Lanxin USV, the vector thrust distribution direction is shown in Figure 6.

When propulsion angle is δ, the thrust distribution values in each direction are as shown in (14).

(14) $$\begin{array}{c} \left\{\begin{array}{c} \tau_{u}=T\cos\delta \\ \tau_{v}=T\sin\delta \\ \tau_{r}=x_{l}T\sin\delta , \end{array}\right. \end{array}$$

where $x_{l}$ is the distance from the center of rotation of the longitudinal arm of the USV to the axial point of the thruster. The effective attack angle of the thruster, $\alpha_{R}$, is a small value with the unit “rad”. Thus, we have $\sin\alpha_{R}\approx \alpha_{R}=\delta$, where the propulsion angle is δ∈[−0.5236rad,0.5236rad]. If $x_{l}>1$; we can think of $\tau_{v}$ as being equal to zero. At the same time, $m_{11}=m-X_{\dot{u}}$, $m_{22}=m-Y_{\dot{v}}$, $m_{23}=mx_{g}-Y_{\dot{r}}$, $m_{32}=mx_{g}-N_{\dot{v}}$, $m_{33}=I_{z}-N_{\dot{r}}$, $d_{11}=-X_{u}$, $d_{22}=-Y_{v}$, $d_{23}=-Y_{r}$, $d_{32}=-N_{v}$, and $d_{33}=-N_{r}$ are defined. Then, (13) can be changed into

(15) $$\begin{array}{c} \left[\left.\begin{array}{c} m_{11}\dot{u}\\ m_{22}\dot{v}+m_{23}\dot{r}\\ m_{32}\dot{v}+m_{33}\dot{r} \end{array}\right]\right.+\left[\left.\begin{array}{c} d_{11}u-m_{22}vr-m_{23}r^{2}\\ d_{22}v-d_{23}r+m_{11}ur\\ m_{22}vu+m_{23}ru+d_{32}v-m_{11}uv+d_{33}r \end{array}\right]\right.=\left[\left.\begin{array}{c} \tau_{u}\\ 0\\ \tau_{r} \end{array}\right]\right. \end{array}$$

One can get (16) by simplifying (15):

(16) $$\begin{array}{c} \left\{\begin{array}{c} \dot{u}=\frac{d_{11}u}{m_{11}}-\frac{m_{22}vr}{m_{11}}-\frac{m_{23}r^{2}}{m_{11}}+\frac{\tau_{u}}{m_{11}}\\ \dot{v}=\frac{(m_{23}m_{22}^{2}-m_{11}m_{22}m_{23}-m_{11}m_{23}-m_{11}m_{22}m_{33}-m_{11}m_{23}m_{32})uv}{m_{22}\left(m_{22}m_{33}-m_{23}m_{32}\right)}+\frac{m_{23}^{2}rv}{\left(m_{22}m_{33}-m_{23}m_{32}\right)}\\ +\frac{(m_{22}m{}_{23}d_{32}+m_{23}m_{32}d_{23}+m_{23}m_{32}d_{22}-m_{22}m_{33}d_{22})v}{m_{22}\left(m_{22}m_{33}-m_{23}m_{32}\right)}+\frac{(m_{33}d_{33}+m_{33}d_{23})r}{\left(m_{22}m_{33}-m_{23}m_{32}\right)}-\frac{m_{23}\tau_{r}}{\left(m_{22}m_{33}-m_{23}m_{32}\right)}\\ \dot{r}=\frac{(m_{11}+m_{11}m_{22}-m_{22}^{2})uv}{m_{22}m_{33}-m_{23}m_{32}}-\frac{m_{22}m_{23}rv}{m_{22}m_{33}-m_{23}m_{32}}-\frac{(m_{22}d_{32}+m_{32}d_{23})v}{m_{22}m_{33}-m_{23}m_{32}}-\frac{(m_{22}d_{33}+m_{32}d_{23})r}{m_{22}m_{33}-m_{23}m_{32}}+\frac{m_{22}\tau_{r}}{m_{22}m_{33}-m_{23}m_{32}}. \end{array}\right. \end{array}$$

**Assumption** **1.** *It is assumed that a USV is symmetrical and the barycenter of the USV coincides with the center of the body-fixed frame. That is to say,* $x_{g}=0$ *,* $Y_{\dot{r}}=0$ *,* $N_{\dot{v}}=0$ *,* $Y_{r}=0$ *, and* $N_{v}=0$ *.*

Based on Assumption 1, (16) can be reduced to

(17) $$\begin{array}{c} \left\{\begin{array}{c} \dot{u}=\frac{m_{22}}{m_{11}}vr-\frac{d_{11}}{m_{11}}u+\frac{1}{m_{11}}\tau_{u}\\ \dot{v}=-\frac{m_{11}}{m_{22}}ur-\frac{d_{22}}{m_{22}}v\\ \dot{r}=\frac{m_{11}-m_{22}}{m_{33}}uv-\frac{d_{33}}{m_{33}}r+\frac{1}{m_{33}}\tau_{r}. \end{array}\right. \end{array}$$

It can be seen that for the vector propulsion of a USV, the three DOF model is an underactuated system. In addition, the results of this theoretical study can also be extended to general vector propulsion ships.

### 4.2. Response Model

In Section 4.1, we concluded that the three DOF model of vector propulsion of a USV is an underactuated system. However, due to the many limitations of underactuated systems, the ship’s underactuation model is used in theoretical studies, in most cases [31,32]. In practical engineering applications, the response model is mainly used for course control and path following.

According to Assumption 1, the mathematical model of planar motion with three DOF can also be expressed as

(18) $$\begin{array}{c} \left\{\begin{array}{c} \left(m+m_{x}\right)\dot{u}-\left(m+m_{y}\right)vr=X_{H}+X_{P}\\ \left(m+m_{y}\right)\dot{v}+\left(m+m_{x}\right)ur=Y_{H}+Y_{P}\\ \left(I_{zz}+J_{zz}\right)\dot{r}=N_{H}+N_{P}, \end{array}\right. \end{array}$$

where $X_{H}$, $Y_{H}$, and $N_{H}$ are the hydrodynamic forces and moments acting on bare hulls. The Taylor series expansion can be expressed as

(19) $$\begin{array}{c} \left\{\begin{array}{c} X_{H}=X\left(u\right)+X_{Hvv}v^{2}+X_{Hvr}vr+X_{Hrr}r^{2}\\ Y_{H}=Y_{Hv}v+Y{}_{Hr}r+Y_{NL}\\ N_{H}=N_{Hv}v+N_{Hr}r+N_{NL}, \end{array}\right. \end{array}$$

where $I_{zz}$ is the moment of inertia.

**Remark** **4.** *The added mass and the added moment of inertia are essentially the same as the acceleration hydrodynamic derivatives. Their mutual correspondence is that* $m_{x}∼-X_{\dot{u}}$ *,* $m_{y}∼-Y_{\dot{v}}$ *,* $J_{zz}∼-N_{\dot{r}}$ *,* $\tau_{u}∼X_{p}$ *,* $\tau_{v}∼Y_{p}$ *, and* $\tau_{r}∼N_{p}$ *.*

For convenience of research, $X_{H}$, $Y_{H}$ and $N_{H}$ needed to be linearized. This so-called linearization means that the USV receives less external disturbance, and its motion is always near the initial equilibrium state. At the same time, all kinds of hydrodynamic terms acting on USV are dominated by linear terms, and the orders of magnitude above the second order are negligible.

Generally, the uniform rectilinear motion of a USV is taken as the initial equilibrium state, and it is assumed that $u=u_{0}$, $v=v_{0}=0$, $r=r_{0}=0$ and $\delta =\delta_{0}=0$. $u_{0}$ is the initial longitudinal velocity of a USV. When a USV is subjected to external interference, the variation in its motion state is Δu, Δv=v, Δr=r, and Δδ=δ, respectively. Then, the motion state of the USV is changed to $u=u_{0}+\Delta u$, $v=v_{0}+\Delta v$, $r=r_{0}+\Delta r$ and $\delta =\delta_{0}+\Delta \delta$.

To preserve the first order small quantities, Δu, *v*, *r* and δ, and ignoring the two order and high order small quantities on the machine, (19) can be simplified to

(20) $$\begin{array}{c} \left\{\begin{array}{c} X_{H}=X\left(u_{0}+\Delta u\right)\\ Y_{H}=Y{}_{Hv}v+Y{}_{Hr}r\\ N_{H}=N_{Hv}+N_{Hr}r, \end{array}\right. \end{array}$$

where $X(u_{0}+\Delta u)$ is the direct resistance of USV. It can be further expressed as

(21) $$\begin{array}{c} X\left(u_{0}+\Delta u\right)=-\frac{1}{2}\rho SC_{t}{\left(u_{0}+\Delta u\right)}^{2}, \end{array}$$

where *S* is the wet area, ρ is the water density, and $C_{t}$ is the total drag coefficient.

(22) $$\begin{array}{c} X\left(u_{0}+\Delta u\right)=-\frac{1}{2}\rho S\left[C_{t0}+{\left(\frac{\partial C_{t}}{\partial \Delta u}\right)}_{u0}\Delta u\right]{\left(u_{0}+\Delta u\right)}^{2}. \end{array}$$

When the speed is $u_{0}$, its total resistance coefficient is $C_{t0}$. The Δu of (22) is linearized as

(23) $$\begin{array}{c} X_{H}=-\frac{1}{2}\rho SC_{t0}u_{0}^{2}-\frac{1}{2}\rho S\left[2C_{t0}u_{0}+{\left(\frac{\partial C_{t}}{\partial \Delta u}\right)}_{u0}u_{0}^{2}\right]\Delta u. \end{array}$$

To define $X_{0}=-\frac{1}{2}\rho SC_{t0}u_{0}^{2}$ and $X_{Hu}=-\frac{1}{2}\rho S\left[2C_{t0}u_{0}+{\left(\frac{\partial C_{t}}{\partial \Delta u}\right)}_{u0}u_{0}^{2}\right]$, $X_{0}$ is used to represent the straight line resistance of the unmanned vehicle in the initial state. Then $X_{H}=X_{0}+X_{Hu}\Delta u$. Thus, (20) can be expressed as

(24) $$\begin{array}{c} \left\{\begin{array}{c} X_{H}=X_{0}+X_{Hu}\Delta u\\ Y_{H}=Y{}_{Hv}v+Y{}_{Hr}r\\ N_{H}=N_{Hv}+N_{Hr}r. \end{array}\right. \end{array}$$

If δ is small, then sinδ=δ and cosδ=1. One can obtain

(25) $$\begin{array}{c} \left\{\begin{array}{c} X_{P}=T\\ Y_{P}=T\delta \\ N_{P}=x_{l}T\delta . \end{array}\right. \end{array}$$

A very important problem to note is that, in the initial state, the resistance of a USV is balanced with the thrust of the thruster (propeller), which means that $X_{0}+X_{P}=0$. Based on the above conditions, (18) can be changed into

(26) $$\begin{array}{c} \left\{\begin{array}{c} \left(m+m_{x}\right)\Delta \dot{u}=X_{u}\Delta u\\ \left(m+m_{y}\right)\dot{v}+\left(m+m_{x}\right)u_{0}r=Y_{Hv}v+Y_{\mathrm{Hr}}r+X_{p}\delta \\ \left(I_{zz}+J_{zz}\right)\dot{r}=N_{Hv}v+N_{Hr}r+x_{l}X_{p}\delta . \end{array}\right. \end{array}$$

It is visible from the upper form that the first equation is decoupled from the second and third equations in linear motion. In other words, the longitudinal motion and the rotation motion of the USV can be considered separately.

**Assumption** **2.** *The influence of longitudinal velocity change is not taken into account, and the external disturbance of the USV received is very weak.*

In practical applications, engineers consider the *r* more than the *v*. $N_{Hv}=N_{v}$, $N_{Hr}=N_{r}$, and $x_{l}X_{p}=N_{\delta }$ are defined, and the third equation of (26) can be reexpressed as

(27) $$\begin{array}{c} \left(I_{zz}+J_{zz}\right)\dot{r}=N_{v}v+N_{r}r+N_{\delta }\delta . \end{array}$$

In order to simplify the problem, it is assumed that the initial state is uniform motion, and all the motion variables have an initial value of zero. Then, v(0)=0, $\dot{v}\left(0\right)=0$, r(0)=0, $\dot{r}\left(0\right)=0$, δ(0)=0, and $\dot{\delta }\left(0\right)=0$. After the Laplace transformation, Equation (27) can be changed into

(28) $$\begin{array}{c} \left(I_{zz}+J{}_{zz}\right)sr\left(s\right)=N_{v}v\left(s\right)+N_{r}r\left(s\right)+N_{\delta }\delta \left(s\right), \end{array}$$

where v(s)=L[v(t)], r(s)=L[r(t)], and δ(s)=L[δ(t)]. The transfer function between the propulsion angle, δ, and the yaw rate, *r*, can be obtained.

(29) $$\begin{array}{c} H\left(s\right)=\frac{r(s)}{\delta (s)}=\frac{K(1+T_{3}s)}{\left(1+T_{1}s\right)\left(1+T_{2}s\right)}, \end{array}$$

where $T_{1}$, $T_{2}$, $T_{3}$ and *K* are corresponding parameters. $T_{1}T_{2}=\left(\frac{L}{U}\right)\frac{\left(m^{\prime }+{m^{\prime }}_{y}\right)\left({I^{\prime }}_{zz}+{J^{\prime }}_{zz}\right)}{C^{\prime }}$, $T_{1}+T_{2}=\left(\frac{L}{U}\right)\frac{-\left(m^{\prime }+{m^{\prime }}_{y}\right){N^{\prime }}_{r}-\left({I^{\prime }}_{zz}+{J^{\prime }}_{zz}\right){Y^{\prime }}_{v}}{C^{\prime }}$, $K=\left(\frac{U}{L}\right)\frac{{N^{\prime }}_{v}{Y^{\prime }}_{\delta }-{N^{\prime }}_{\delta }{Y^{\prime }}_{v}}{C^{\prime }}$, $KT_{3}=\frac{\left(m^{\prime }+{m^{\prime }}_{y}\right){N^{\prime }}_{\delta }}{C^{\prime }}$, $C^{\prime }=Y_{v}^{\prime }N_{r}^{\prime }-\left[Y_{r}^{\prime }-\left(m^{\prime }+m_{x}^{\prime }\right)\right]N_{v}^{\prime }$, where $m^{\prime }$ is the normalization of *m*. Similarly, $m_{x}^{\prime }$, $m_{y}^{\prime }$ and $I_{zz}^{\prime }$, etc. use superscripts to represent their normalization. Using the Laplace inverse transform, Equation (29) can be transformed into a linear response equation in the time domain:

(30) $$\begin{array}{c} T_{1}T_{2}\ddot{r}+\left(T_{1}+T_{2}\right)\dot{r}+r=K\left(\delta +T_{3}\dot{\delta }\right). \end{array}$$

It is also important to note that if a USV has the characteristic of course stability, $C^{\prime }>0$; if the USV does not have the characteristic of course stability, $C^{\prime }<0$; $C^{\prime }≅0$ is called the critical stability. Therefore, $C^{\prime }$ is the number of stability criteria. The nonlinear change of $C^{\prime }$ was proposed by Nomoto, which is expressed as

(31) $$\begin{array}{c} C^{\prime }=C_{0}^{\prime }+\varpi r^{2}, \end{array}$$

where $C_{0}^{\prime }$ represents the value of $C^{\prime }$ when r=0. ϖ is a newly introduced variable.

Substituting (31) into (30), we obtain

(32) $$\begin{array}{c} {\left(\frac{L}{V}\right)}^{2}\left(m^{\prime }+{m^{\prime }}_{y}\right)\left({I^{\prime }}_{zz}+{J^{\prime }}_{zz}\right)\ddot{r}+\left(\frac{L}{V}\right)\left[\left(m^{\prime }+{m^{\prime }}_{y}\right){N^{\prime }}_{r}-\left({I^{\prime }}_{zz}+{J^{\prime }}_{zz}\right){Y^{\prime }}_{v}\right]\dot{r}+\left({C^{\prime }}_{0}+\varpi r^{2}\right)r=\\ \left(\frac{L}{V}\right)\left({N^{\prime }}_{v}{Y^{\prime }}_{\delta }-{N^{\prime }}_{\delta }{Y^{\prime }}_{v}\right)\delta +\left(m^{\prime }+{m^{\prime }}_{y}\right){N^{\prime }}_{\delta }\dot{\delta }. \end{array}$$

The two sides of (32) are divided by $C_{0}^{\prime }$, and one can obtain

(33) $$\begin{array}{c} {\left(\frac{L}{V}\right)}^{2}\frac{\left(m^{\prime }+{m^{\prime }}_{y}\right)\left({I^{\prime }}_{zz}+{J^{\prime }}_{zz}\right)}{{C^{\prime }}_{0}}\ddot{r}+\left(\frac{L}{V}\right)\frac{\left(m^{\prime }+{m^{\prime }}_{y}\right){N^{\prime }}_{r}-\left({I^{\prime }}_{zz}+{J^{\prime }}_{zz}\right){Y^{\prime }}_{v}}{{C^{\prime }}_{0}}\dot{r}+\frac{{C^{\prime }}_{0}+\varpi r^{2}}{{C^{\prime }}_{0}}r=\\ \left(\frac{L}{V}\right)\frac{{N^{\prime }}_{v}{Y^{\prime }}_{\delta }-{N^{\prime }}_{\delta }{Y^{\prime }}_{v}}{{C^{\prime }}_{0}}\delta +\frac{\left(m^{\prime }+{m^{\prime }}_{y}\right){N^{\prime }}_{\delta }}{{C^{\prime }}_{0}}\dot{\delta }. \end{array}$$

Meanwhile, $\alpha =\frac{n}{{C^{\prime }}_{0}}$ is defined, and then (33) can be simplified as

(34) $$\begin{array}{c} T_{1}T_{2}\ddot{r}+\left(T_{1}+T_{2}\right)\dot{r}+r+\alpha r^{3}=K\delta +KT_{3}\dot{\delta }. \end{array}$$

The nonlinear influence is embodied by $\alpha r^{3}$, and α is the newly introduced constant. In practical applications, (34) is often simplified to a first order form. That is

(35) $$\begin{array}{c} T\dot{r}+r+\alpha r^{3}=K\delta . \end{array}$$

This is Norrbin nonlinear response model used in the field of ship motion control [33,34]. Through the study of this paper, we know that the response model of the class of vector propulsion ships still conforms to the classical Norrbin model. Equation (35) can be reexpressed as $T\dot{r}+r=K\delta$ plus $\alpha r^{3}$. $T\dot{r}+r=K\delta$ is the classic Nomoto model [35,36].

When the USV is conducting the turning test, $\dot{r}=0$, and $\dot{\delta }=0$. (35) can be simplified as

(36) $$\begin{array}{c} r+\alpha r^{3}=K\delta . \end{array}$$

This means that in the case of a known *K*, the nonlinear term coefficient, α, can be fitted through a series of turning tests.

**Remark** **5.** *During the turning test, ideally, an propulsion angle will correspond to a constant value of r. However, under the influence of various conditions, such as external disturbance, the value of r fluctuates. So when fitting the α value, we need to calculate an average r value.*

## 5. Identification and Verification

### 5.1. Identification

#### 5.1.1. Three DOF Model

(1) Data: because the data of the zig-zag model is more able to exert the manoeuvre characteristics of the USV, it is used to identify the three DOF underactuated model. Of course, we mainly used *u*, *v*, *r*, *n* and δ in the zig-zag test.
(2) $\tau_{u}$ and $\tau_{r}$: based on the modeling of the thruster thrust in the Section 2, the real-time $\tau_{u}$ and $\tau_{r}$ were calculated based on the rotating speed of the propeller and the propulsion angle.
(3) Based on the sampling time of 0.02 seconds, $\dot{u}$, $\dot{v}$ and $\dot{r}$ were calculated.
(4) Recursive least squares method was used to identify the parameters of underactuated model.

The results of the identification were as follows:

(37) $$\begin{array}{c} \left\{\begin{array}{c} \dot{u}=1.065245vr-0.3197455u+0.000377\tau_{u}\\ \dot{v}=-0.938753ur-3.5v\\ \dot{r}=-0.041187uv-5.407752r+0.000238\tau_{r}. \end{array}\right. \end{array}$$

#### 5.1.2. Response Model

**Remark** **6.** *In order to improve the identification accuracy, we first identified the Nomoto model. Then, the nonlinear parameter, α, was fitted through a series of turning test data.*

(1) In the zig-zag test, *r* and δ were used to identify the Nomoto model. Then, the propulsion angles, δ, of four sets of turning tests and the corresponding average, *r*, were used to fit α.

(2) The recursive least squares method was used to identify the Nomoto model, and the method of data fitting was used to obtain α.

The identification result from the Nomoto model was

(38) $$\begin{array}{c} 0.332\dot{r}+r=0.707\delta . \end{array}$$

That is to say, K=0.332 and T=0.707. Based on this, Equation (35) was rewritten as

(39) $$\begin{array}{c} 0.332\dot{r}+r+\alpha r^{3}=0.707\delta . \end{array}$$

The result of parameter fitting was α=1.102. The final identification result of the Norrbin model was

(40) $$\begin{array}{c} 0.332\dot{r}+r+1.102r^{3}=0.707\delta . \end{array}$$

#### 5.1.3. Servo Model

(1) Data: the target propulsion angle $\delta_{d}$ (input) and the actual propulsion angle δ (output) are derived from the zig-zag test data.

(2) The recursive least squares method was used to identify the servo model.

The identification result of servo model was

(41) $$\begin{array}{c} \frac{\delta }{\delta_{d}}=\frac{1.95}{s^{2}+2.38125s+1.95}e^{-t}. \end{array}$$

**Remark** **7.** *The upper control computer output a control command every 0.02 seconds through the timer. The servo system took about 1 second from receiving the instruction to actually start to perform rotation. So a 1 s delay was added to the servo model.*

### 5.2. Verification

In this section, the identified models were simulated by the turning test and the zig-zag test, and then the results were compared with the actual data, to verify the feasibility and correctness of the modeling and identification results.

#### 5.2.1. Three DOF Model

**Figure 7:**
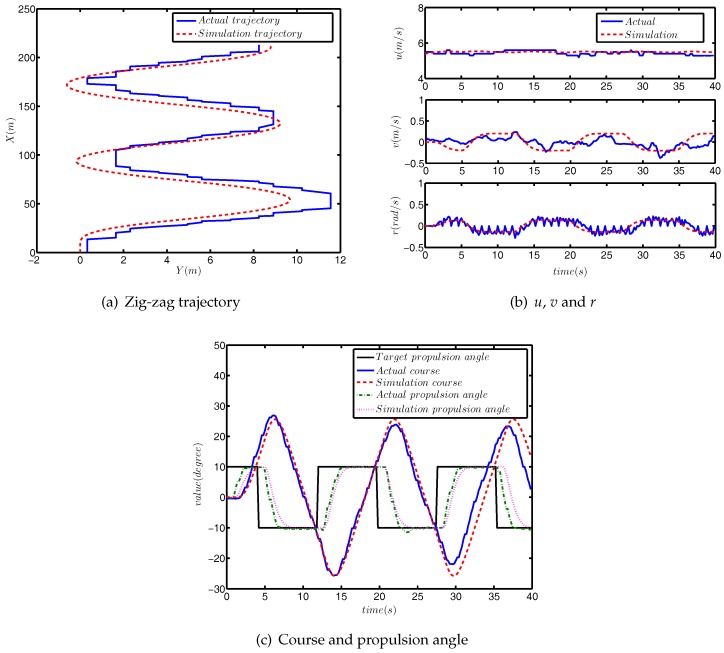
The comparison results of the zig-zag test.

First, we carried out the zig-zag test simulation experiment for model (37), and the results of the comparison are shown in Figure 7.

Figure 7a depicts the actual trajectory and the simulation trajectory. It can be seen from Figure 7a that the maximum lateral error between the actual data and simulated data is about 1.5 m, which accounts for 12.5% of the total lateral distance. The maximum longitudinal error is about 10 m, accounting for 4.5% of the total longitudinal distance. Due to the existence of external disturbance, the USV will have some drift, so the proportion of lateral error is greater than that of longitudinal, which is also reasonable. Figure 7b shows the comparison of *u*, *v* and *r* in the actual and simulated data. It can be clearly seen from the picture that the results of *u* and *r* basically coincided, and the simulation trend, *v*, was also the same. It is obvious from Figure 7c that the maximum difference between the actual and simulated course angles is about 3°. In addition, in the later stage of simulation, compared with the actual data, the phase difference between the simulated course and propulsion angle is caused by external disturbance.

**Remark** **8.** *First of all, external disturbances include the actual wind, waves, and currents, and the currents generated by the USV’s own motion. To be more precise, in a stable sea state, the adverse effects of the currents generated by USV’s own motion may be greater than weather. Secondly, the sensors’ own factors can also cause measurement noise. Due to the above two points, the measured values of the yaw rate had a jagged appearance.*

**Remark** **9.** *Although the experimental environment is selected in a relatively calm sea state, the disturbance of wind, waves and currents still exists. Compared with large merchant ships, the USV has a smaller volume and is more sensitive to the external environment, especially lateral disturbance. Therefore, in the result of model validation, the error of v is larger than that of u and r.*

**Remark** **10.** *In the process of model identification, a certain modeling error is allowed. The main reasons are as follows:*

*(1)*  *In terms of modeling theory, the derivation of the model is based on various assumptions and simplification. That is to say, it is difficult to fully reflect the characteristics of a USV with the mathematical model.*
*(2)*  *During the voyage, due to the influences of sea condition, operation and various factors, the structure and parameters of a USV will change. In addition, when designing various USV controllers, designers take the uncertainty of the model parameters or structure into account [37].*
*(3)*  *For the real ship, even though the related field experiments are carried out in a relatively calm sea area, the external interference is inevitable.*

**Figure 8:**
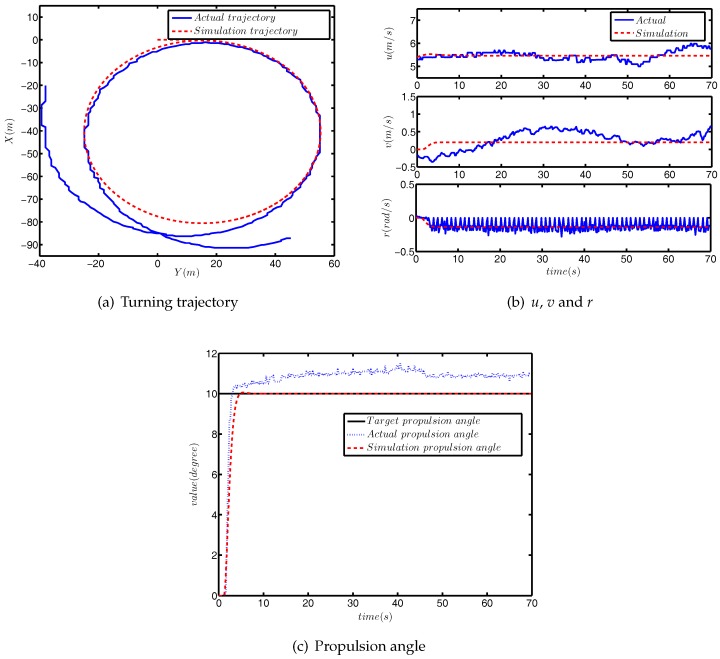
The comparison results of the turning test.

The comparison between simulation turning test and actual turning test is shown in Figure 8.

As can be seen from Figure 8a, the difference between the simulated radius of rotation and the actual radius was approximately 1.5 m, which accounts for 3.8% of the actual radius. The comparison results of *u*, *v* and *r* are provided in Figure 8b, and the differences between the simulation value and actual value were within a reasonable range. Figure 8c shows the response curves of the propulsion angle. The target propulsion angle was 10°, and the actual target propulsion angle was about 11°, which is due to external disturbance.

#### 5.2.2. Response Model

**Figure 9:**
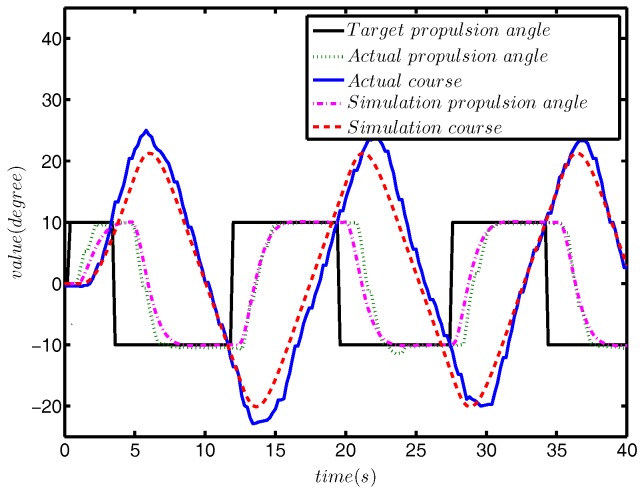
The comparison results of the zig-zag test.

**Figure 10:**
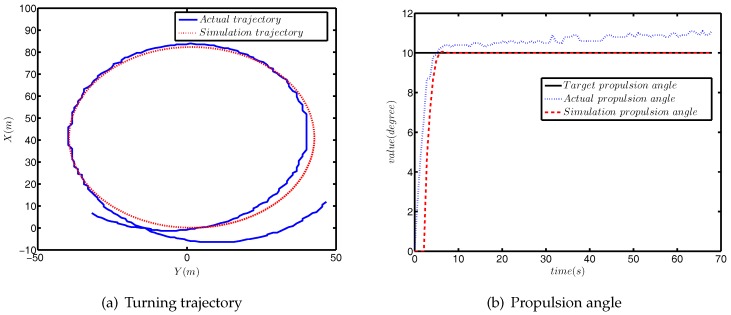
The comparison results of the turning test.

In this section, we verify the correctness of the identification of the response model through the simulation and actual comparison. The comparison results of the zig-zag test are provided in Figure 9, and the comparison results of the turning test are plotted in Figure 10.

Figure 9 shows that as with the verification of the three DOF model, there was a certain phase difference in the course. Meanwhile, the maximum course error was approximately 3°, accounting for 7.5% of the total course angle. Figure 10 displays that the difference between the simulation trajectory and the actual trajectory was very small. The above two comparison results are sufficient to prove the correctness of the identification results of the final response model.

**Remark** **11.** *The validation of the thruster thrust model and servo model were completed in the verification of three DOF model, so there was no need to verify them separately.*

## 6. Course Keeping Field Experiment

On the basis of the obtained thruster servo model and response model, the numerical simulation and field experiment of course keeping are given to further verify the results of the theoretical research.

### 6.1. Numerical Simulation

In this paper, the proportional-derivative (PD) course keeping controller was used for the simulation and field experiments. At the same time, the PD control was compared with the proportional-integral-derivative (PID) control to verify the role of the integral term in the course control. The expression of the PID control is as follows [38]:

(42) $$\begin{array}{c} \delta =-K_{p}\left(\psi -\psi_{d}\right)-K_{i}\int \left(\psi -\psi_{d}\right)-K_{d}{\left(\psi -\psi_{d}\right)}^{\prime }, \end{array}$$

where $K_{p}$, $K_{i}$ and $K_{d}$ are the three positive control parameters, and $\psi_{d}$ is the target course. The parameters of the PID control are $K_{p}=0.7$, $K_{i}=0.00095$ and $K_{d}=1$. The parameters of the PD control are $K_{p}=0.7$ and $K_{d}=1$. The gains of the PD controller were manually adjusted by the manipulator. The specific steps were as follows: (1) the control gain, $K_{p}$, was gradually adjusted until the course appeared to have equal amplitude oscillation; and (2) $K_{d}$ was slowly adjusted from zero to optimize the control effect (this is also a Ziegler–Nichols tuning method. Of course, this is done manually). Meanwhile, the integral of time-weighted absolute error (ITAE) index was used to quantify the control accuracy of the two algorithms:

(43) $$\begin{array}{c} ITAE=\int_{0}^{t}t\left|\psi -\psi_{d}\right|dt. \end{array}$$

**Remark** **12.** *In actual ship motion control, PD control is often used instead of PID control. The reason for this is that the marine environment is often very bad, and external disturbance is inevitable. Persistent disturbance often causes saturation of the integral, so the integral term should be used with caution.*

**Figure 11:**
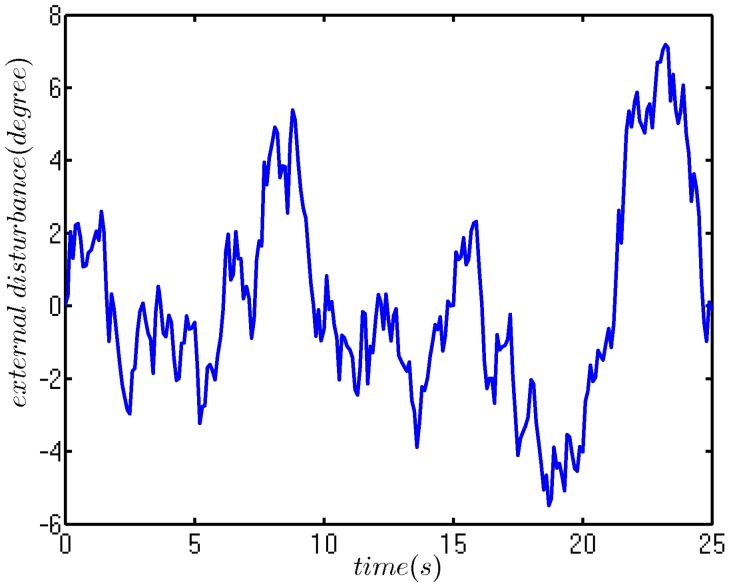
External disturbance curve.

The white noise is used to drive the transfer function, $\frac{0.42s}{s^{2}+036s+0.37}$, to describe the external disturbance. The disturbance curve is depicted in Figure 11.

**Figure 12:**
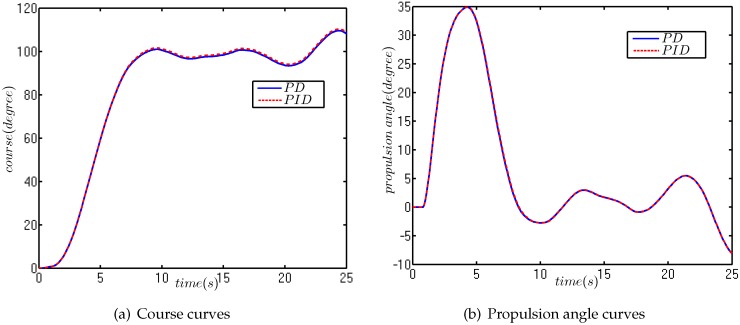
The numerical simulation of course keeping.

The initial course is 0°, and the target course is 100°. The simulation results are plotted in Figure 12, and the ITAE comparison results are shown in Table 3.

**Figure 13:**
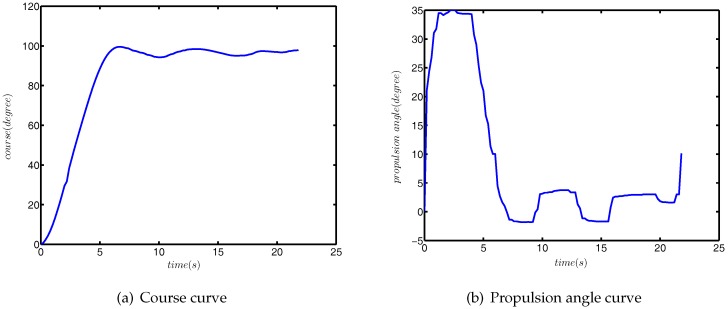
The field experiment for course keeping.

Figure 13 shows that the control effects of PID and PD were almost the same, and both were able to maintain the course of a USV near the target course. In addition to that, the ITAE indexes of PID and PD were 37.98 and 38.6 respectively. The introduction of an integral item in the same case of $K_{p}$ and $K_{d}$ played a positive role, but the value of this integral term was very small. Therefore, the integral item can be introduced in actual course control, but there is need to be cautious.

### 6.2. Field Experiment

The final purpose of modeling and identification is to carry out practical applications. In this subsection, we conduct course keeping in a field experiment to compare with the numerical simulation.

(1) Experimental sea area: nearby waters (longitude:121.5548, latitude: 38.8612)
(2) Sea state: three-level to four-level marine conditions.
(3) Weather: the sea breeze was about three-levels north wind.
(4) The driving speed of USV was kept at around 10 knots.

The initial course of the USV was 0°, and the target course was 100°. The results of the field experiment are shown in Figure 13.

The actual performances were very similar to the simulation results of the numerical simulation. The course angle was maintained near the target value, and the propulsion angle was constantly changing to resist the external disturbance. In addition, due to the course keeping, the field experiment was carried out in three- to four-level marine conditions, the initial propulsion angle was not 0°, in order to maintain stability. So, there appears to be a jump at the beginning of propulsion angle curve.

## 7. Conclusions

In this paper, a complete set of schemes has been proposed for vector propulsion of a USV, from data acquisition, model establishment, parameter identification, result verification, and finally, a real ship field experiment. Linking theory with practice is the greatest advantage of this paper. First, based on the hypothesis and simplification, the thrust model, thruster servo model, three DOF model and response model re established respectively. It was proven that vector propulsion of a USV belongs is an underactuated system, and its response model is in accordance with the Norrbin model structure. Then, the parameters of three DOF model, response model and thruster servo model were identified and verified. Finally, based on the thruster servo model and the response model, numerical simulation and real ship field experiment for course keeping were carried out. In future research, the trajectory tracking controller will be designed.

## Figures and Tables

**Table 1:** Specific Parameters of the Lanxin Unmanned Surface Vehicle (USV).

Item	Value
Length between perpendiculars	7.02 m
Breadth	2.60 m
USV speed (max)	35 kn
Draft (full load)	0.32
Block coefficient	0.6976
Displacement (full load)	2.73 m^3^
Rudder area	0.2091 m^2^
Propulsion angle (max)	35 degrees
Distance Between gravity and center	0.35 m
Pitch ratio	0.3
Disk surface ratios	0.516
Diameter of the propeller	0.46 m

**Table 2:** The coefficients of the propeller thrust coefficient expression.

Item	θ=0.5	θ=0.8	θ=1.1
$A_{00}$	−0.1677	−0.0517	−0.2191
$A_{01}$	0.1747	−0.0315	0.3013
$A_{02}$	−0.6720	−0.5822	−0.7309
$A_{10}$	0.8042	0.5853	−0.8502
$A_{20}$	−0.1437	−0.1026	−0.1080
$A_{30}$	0	0	0
$A_{11}$	−0.8853	−0.3381	−1.0738
$A_{12}$	0.9130	0.6654	0.9908
$A_{21}$	0.3422	0.1417	0.3481
$A_{22}$	−0.3276	−0.2215	−0.3322

**Table 3:** ITAE index.

Item	Value
PID	37.98
PD	38.6

## Abbreviations

The following abbreviations are used in this manuscript:

USV	unmanned surface vehicle
DOF	degree of freedom
MMG	manoeuvring mathematical model group
EKF	extended Kalman filter
GPS	global position system
IIC	inter integrated circuit
NMEA	national marine electronics association
CAN	controller area network
MEMS	microelectro mechanical systems
MFC	microsoft foundation classes
PID	proportional integral derivative
PD	proportional derivative
ITAE	the integral of time-weighted absolute error
